# Antimicrobial Potential of Food Lactic Acid Bacteria: Bioactive Peptide Decrypting from Caseins and Bacteriocin Production

**DOI:** 10.3390/microorganisms9010065

**Published:** 2020-12-29

**Authors:** Stefano Nebbia, Cristina Lamberti, Giuliana Lo Bianco, Simona Cirrincione, Valerie Laroute, Muriel Cocaign-Bousquet, Laura Cavallarin, Maria Gabriella Giuffrida, Enrica Pessione

**Affiliations:** 1 Laboratory of Microbial and Applied Biochemistry-Department of Life Sciences and Systems Biology, Università di Torino, Via Accademia Albertina 13, 10123 Torino, Italy; stefano.nebbia@ispa.cnr.it (S.N.); giuliana.lobianco@gmail.com (G.L.B.); enrica.pessione@unito.it (E.P.); 2 Consiglio Nazionale delle Ricerche-Istituto di Scienze delle Produzioni Alimentari, Largo Braccini, 10095 Grugliasco, Italy; cristina.lamberti@ispa.cnr.it (C.L.); laura.cavallarin@ispa.cnr.it (L.C.); gabriella.giuffrida@ispa.cnr.it (M.G.G.); 3 Laboratoire d’ingénierie des systèmes biologiques et des procédés, Université de Toulouse, CNRS, INRA, INSA 135 Avenue de Rangueil, 31077 Toulouse, France; vlaroute@insa-toulouse.fr (V.L.); cocaign@insa-toulouse.fr (M.C.-B.)

**Keywords:** antimicrobial peptides, casein, lactic acid, mass spectrometry, Sakacin, Nisin Z. *Lactobacillus sakei* I151, *Lactococcus lactis*, *Lactobacillus rhamnosus* 17D10, *Lactobacillus helveticus* 4D5

## Abstract

Lactic acid bacteria (LAB) potential in the food industry and in the biotechnological sector is a well-established interest. LAB potential in counteracting especially food-borne infections has received growing attention, but despite being a road full of promises is yet poorly explored. Furthermore, the ability of LAB to produce antimicrobial compounds, both by ribosomal synthesis and by decrypting them from proteins, is of high value when considering the growing impact of multidrug resistant strains. The antimicrobial potential of 14 food-derived lactic acid bacteria strains has been investigated in this study. Among them, four strains were able to counteract *Listeria monocytogenes* growth: *Lactococcus lactis* SN12 and *L. lactis* SN17 by high lactic acid production, whereas *L. lactis* 41FLL3 and *Lactobacillus sakei* I151 by Nisin Z and Sakacin P production, respectively. Strains *Lactococcus lactis MG1363*, *Lactobacillus rhamnosus 17D10* and *Lactobacillus helveticus* 4D5 were tested and selected for their potential attitude to hydrolyze caseins. All the strains were able to release bioactive peptides with already known antimicrobial, antihypertensive and opioid activities. These features render these strains or their bioactive molecules suitable for use in food as biocontrol agents, or as nutraceutical supplements to treat mild disorders such as moderate hypertension and children insomnia. These results highlight once again that LAB potential in ensuring food safety, food nutraceutical value and ultimately in favoring human health is still underexplored and underexploited.

## 1. Introduction

Lactic acid bacteria (LAB) are GRAS (generally recognized as safe) organisms that have been employed as starters since the last century in different food sectors such as the dairy industry, breweries, wine-making and many others (fermented sausages and sauerkrauts production) [1]. Their probiotic role as health-promoting microorganisms, firstly perceived at the beginning of the 20th century by the Russian biologist Metchnikoff [2] has been extensively investigated in the last decades and a huge number of LAB species have been included in the probiotic list [3]. Probiotics can control almost all physiological functions of the host organism among which the most important are nutritional status [4], metabolism [5], immunity [6], mental health and mood [7]. More recently, LAB have found application also as cell factories for bioconversions. As an example, waste conversion into lactic acid has been used as the building block for polylactide biodegradable polymers (PLA) production [4].

However, a well-known but still underexploited role is their ability to control food spoilage and food-borne infections. Regarding spoilage, LAB have been employed to extend the shelf-life of food [8], when suitably tested to avoid reciprocal antagonistic action with starter strains when fermented food is concerned. Attempts were also made to counteract food-borne infections such as those caused by *Staphylococcus aureus* [9] and *Listeria monocytogenes* [10]. More recently, LAB have attracted researcher’s attention as alternative treatment to antibiotic therapy [11]. Antibiotic resistance is considered a major threat to public health (World Health Organization 2014) since it is widespread in pathogenic, commensal and food bacteria. The growing impact of multidrug resistant (MDR) strains originating from selective pressure by unnecessary antibiotic abuse in the past 60 years, requires the urgent finding of new antimicrobial drugs [12]. The prolonged antibiotic treatments on farm animals is also responsible of antibiotic resistance among cheese starters or meat biocontrol strains [13]. Together with the emergence of severe food-borne infections such as those caused by *Listeria monocytogenes* and *Staphylococcus aureus* [14], this makes the food ecological niche an ideal habitat for horizontal gene transfer promoting acquisition of resistance genes by pathogens. Therefore, a solution should urgently be found to ensure food safety for the consumers [15].

Several strategies have been proposed in the past two decades to respond to the need of new antibacterial agents. From one side, a bacteriostatic and bactericidal effect can be obtained by the use of inorganic and organic compounds such as metals [16], surfactants [17] and plant essential oils [18], however these molecules are not always suitable for food use. From another side, the use of living organisms, although sometimes controversial, such as bacteriophages [19,20] and competing probiotic strains [21] has been suggested as well. In addition, quorum-quenchers have been proposed for fighting infections caused by both Gram negative [22] and Gram-positive [23] bacteria, since in some cases an alternative to killing bacteria is to prevent the production of toxins and other virulence factors that are synthesized under quorum sensing control.

However, a current winning strategy is the use of bacterial derived compounds such as metabolic end-products and bacteriocins [24]. The microbial world has a great potential in producing antibacterial compounds which differ deeply in their structure, including catabolic end-products such as solvents (acetone, butanol, and ethanol) and acids (lactic, acetic, formic, and butyric) but also peptides, proteins and enzymes [21]. Screening natural microbial strains to find the ones able to produce antimicrobial molecules is a promising strategy. In particular, lactic acid bacteria (LAB) are good candidates for finding molecules preventing food-borne infections since they have a long evolutionary history in the food ecological niche in which fighting competitive species is pivotal for surviving. A huge number of either bacteriostatic or bactericidal compounds from LAB have been described [1]. An emblematic example is *Lactobacillus reuteri* that can synthesize at least three molecules interfering with bacterial growth: reuterin, reuterocyclin and reutericin. Reuterin has a molecular mass lower than 100 Da and exerts its antimicrobial activity thanks to the complex formed with gut amines [25]. Reuterocyclins are small hydrophobic compounds with a molecular mass of 349 Da that exhibits a broad inhibitory spectrum against *Bacillus subtilis*, *Bacillus cereus*, *Enterococcus faecalis*, *Listeria innocua*, and *Staphylococcus aureus*. On the latter microorganisms, a bactericidal mode of action triggering cell lysis has been demonstrated [26]. Reutericin 6 is a bacteriocin-like proteinaceous molecule with a molecular weight of 2.700 Da and its amino acid analysis reveals a composition characterized by 67% of hydrophobic and polar neutral amino acids [27].

Bacteriocins are small peptides or proteins, secreted or surface-bound, active at very low concentration (nanomolar). They are highly specific and generally can kill the target bacteria by acting at the cell surface (membrane or cell-wall) level [14]. Some bacteriocins are involved in the dissipation of the proton gradient across the bacterial cytoplasmic membrane, other inhibit the biosynthesis of the cell wall or also create “pores” which cause loss of metabolites [28,29]. In spite of the fact that some of them have been used for about 50 years as food additives (to prevent spoilage and food borne infections) only few cases of resistance against bacteriocins have been reported [30,31].

LAB are high performing producers of these molecules [11]: nisin A (the first patented bacteriocin) has been isolated in 1928 [32] and also pediocin, enterocin, sakacin, lactococcin, helveticin, reutericin, lactacine, salivaricine, mutacine, gassericine represent examples of LAB products [33]. The present limit of bacteriocin use in human infection control lies in the fact that most of these molecules are peptides, hence they can be hydrolyzed during the gastric transit by endogenous peptidases. Several attempts have been made to protect bacteriocins from degradation, and, among these, the resistance to proteases is one of the most promising [34]. However, these molecules can be purified and encapsulated to reach the lower gastrointestinal tract intact, where they can exert a beneficial activity both for controlling gut infections and being absorbed and having a systemic action.

In parallel to bacteriocins, other interesting antimicrobial molecules released by LAB are not of metabolic origin but rather the result of a proteolytic action on different proteins, generally present in food [35]. Casocidin (αs2-casein-derived peptide), isracidin, consisting of the first 23 amino acids of αs1-casein, and the k-casein-derived kappacin display antimicrobial action against *S. aureus* but also towards *Bacillus*, *L. monocytogenes*, *Escherichia coli*, *Klebsiella*, *Salmonella* and *Pseudomonas* [36,37]. LAB are equipped with a very efficient proteolytic system (which includes extracellular proteases, surface peptidases, transporters and intracellular oligopeptidases) and they can liberate encrypted antibacterial peptides from food proteins such as milk proteins (casein, alpha-lactalbumin, lactoferrin, lactadherine, lactoglobulines), gluten, soya and bovine hemoglobin [1]. The mode of action of these peptides is based upon a strongly positively charged domain that can interact with teichoic acids (in Gram-positive bacteria) or LPS (in Gram-negative bacteria) [38]. Later the hydrophobic moiety of the peptide can bind to bacterial membranes, dissipating the proton gradient and causing membrane depolarization (like the antibiotics polimixin B and colistin) and cell lysis, similarly to what happens with bacteriocins [39]. Other mechanisms, for instance a synergic action with host innate immunity and a metabolic perturbation, were also described [40]. The stability of these peptides in human blood render them promising anti-infection agents.

The aim of the present investigation was to test the antimicrobial potential of different food-isolated LAB by detecting antimicrobial activities against *L. monocytogenes*. In parallel, negative strains (not able to counteract Listeria growth) were tested for their capability to decrypt antibacterial peptides from alpha, beta and kappa casein. The experimental plan concerned: (i) the evaluation of lactate dehydrogenase activity, (ii) the characterization and purification of bacteriocins, (iii) the evaluation of bacterial ability to hydrolyze milk proteins and release antimicrobial peptides, characterized by mass spectrometry. The final objective is to have information on the potential antimicrobial and proteolytic attitude of different LAB strains in order to optimize methods to obtain molecules possibly useful in counteracting food-borne infections afigurend food spoilage.

## 2. Materials and Methods

### 2.1. Chemicals and Instruments

All chemicals were from Merck KGaA (Saint Luis, MI, USA). VersaMax Microplate and SoftMax Pro were from Molecular Devices (San Jose, CA, USA). Bradford assay, Mini-PROTEAN Tetra Cell and Mini-Protean Tricine precast gels were from Bio-Rad (Hercules, CA, USA). Plus DNA Ladder and PageRuler Low range unstained protein ladder were from Life Technologies (Carlsbad, CA, USA). Stericup filters were from Millipore (Burlington, MA, USA). HiTrap SP FF cation exchange column and HiTrapOctyl FF hydrophobic interaction column were from GE Healthcare (Little Chalfont, Buckinghamshire, UK). Vivacel 250 membrane filter were from Sartorius (Gottinga, Germany). Ultraflex III MALDI-TOF/TOF instrument and Flex Analysis software were from Bruker Daltonik GmbH (Bremen, Germany).

### 2.2. Isolation of LAB from Food Samples

Fourteen LAB, isolated from food samples, were grown on both MRS (allowing growth of lactobacilli) and M17 (allowing growth of lactococci) media, both at 30 °C and 37 °C and were identified by the Crystal ANR and GP (BBL) (Thermo Fisher Scientific, Waltham, MA, USA) based on fluorogenic and chromogenic analysis of metabolic products. Twelve bacterial strains were isolated from cheese. They belonged to the following species: *Lactobacillus. acidophilus* (4 strains), *Lactobacillus. casei* (3 strains), *Lactobacillus hilgardii* (1 strain) *Lactobacillus helveticus* (1 strain 4D5), *Lactobacillus rhamnosus* (1 strain 17D10) and *Lactococcus lactis* (2 strains). Further two strains (*Lactobacillus.sakei* I151 and *Lactococcus lactis* 41FLL3) were isolated from fermented sausages. We also considered a reference strain (*L. lactis* MG1363) belonged to the culture collection of the Laboratoire d’Ingénierie des Systèmes Biologiques et des Procédés (LISBP), Toulouse. The strains were maintained in suitable culture medium at −20 °C in 0.5 mL aliquots with 0.5 mL of 40% (*v/v*) glycerol.

### 2.3. Selection of Strains Producing Antimicrobial Molecules

All isolated strains, potentially producing antimicrobial compounds, were tested against *L. monocytogenes* using in liquid assay. Aliquots (50 µL) of cell free supernatants obtained by centrifugation (4000× *g*, 20 min, 4 °C) of LAB cultures grown for 18 h in closed 250 mL screw cap bottles in either MRS or M17 broth were added to 1 mL of a freshly inoculated *L. monocytogenes* culture and incubated at 37 °C for 24 h. The antimicrobial activity was evaluated by observing the OD_600_ decrease, using the OD_600_ value of *L. monocytogenes* alone as reference. The inhibition percentage was calculated through the formula: 1- (OD_600_ fraction/ OD_600_ *L. monocytogenes*) × 100). In order to confirm the proteinaceous nature of the antimicrobial molecules, 20 μL of proteinase K (buffered aqueous glycerol solution ≥ 10 mg/mL) were incubated with 50 µL of supernatants for 1 h at 37 °C. Then, the samples were incubated for 10 min at 70 °C to inactivate the enzyme. The treated samples were processed as the other samples for antimicrobial activity.

### 2.4. Detection of Lactate Dehydrogenase (LDH) Activity

Ten mg of bacteria were collected by centrifugation (4000× *g*, 20 min, 4 °C), washed 3 times with 0.2% KCl, and mixed with 0.5 g of glass beads and 1 mL of extraction buffer (70% Tris/HCl 1 M pH 7.5, 23% glycerol, 7% MgCl_2_ 50 mM and 170 µL DTT 300 mM). Bacterial lysis was performed by alternating six cycles of 30 s Vortex (6.5 rpm) with 1 min on ice. Bacterial lysate diluted in water (5 and 30 times, respectively for lactobacilli and lactococci) was used to evaluate the lactate dehydrogenase (LDH) activity. Samples (80 µL) were mixed with 110 µL of enzymatic buffer (46% Tris/HCl 400 mM, 18% MgCl_2_ 50 mM, 18% NADH 6 mM and 18% Fructose 1–6 bisphosphate 30 mM) and 10 µL of 400 mM pyruvate as substrate. LDH activity was measured as the decrease of NADH peak at 340 nm in 96 wells microtiter plates and SoftMax Pro was used for data processing. The activity was expressed as Units (U)/mL of cell lysate/g protein (1 U = 1 µmol of substrate hydrolyzed per minute). Total protein content from bacterial lysate were quantified by the Bradford assay [41], using BSA as the standard.

### 2.5. Identification of the nisZ Gene in L. lactis 41FLL3 by PCR

*L. lactis* 41FLL3 was grown in M17 medium and cells were collected at the log phase by centrifugation (4000× *g*, 20 min, 4 °C) and resuspended in water. Based on the work of Sunita et al. (2012) [42], specific primers (Forward: 5′-ATGAGTACAAAAGATTTTAACTTGG-3′ and Reverse: 5′-TTATTTGCTTACGTGAATACTACA-3′) were used to amplify *nisZ* gene using a Bio-Rad thermal cycler. Thermocycling conditions were as follows: initial cell lysis for 16 min at 95 °C then 34 cycles of 15 sec at 95 °C, 15 sec at 55 °C and 30 sec at 72 °C. The final cycle was followed by 5 min of incubation at 72 °C. Ultrapure water was used as the negative control. After reaction, 6 µL of sample was loaded in 2.5% (*w/v*) agarose gel in TBE (Tris Borate, EDTA) and 1Kb Plus DNA Ladder was used as the marker.

### 2.6. Purification and Quantification of Nisin Z and Sakacin P

At the beginning of the stationary phase, 200 mL of supernatants from *L. sakei* I151 and *L. lactis* 41FLL3 culture was recovered. Bacteria were removed by centrifugation (4000× *g*, 20 min, 4 °C) followed by filtration in stericup 0.22 μm filters.

Nisin Z purification: Supernatants obtained from *L. lactis* 41FLL3 cultures were diluted with 400 mL of 50 mM lactic acid pH 3 and loaded on a 5 mL HiTrap SP FF cation exchange column using a flow rate of 4 mL/min. The column was washed with 50 mL lactic acid pH 3 to remove non-specific contaminants. The elution was performed with 50 mL lactic acid pH 3 added with a NaCl gradient increasing from 0.2 M to 1 M (10 mL for all the NaCl concentration; gradient step 0.2 M). Two mL fractions were collected and their Nisin Z content was evaluated by both OD_215_ measurement and the previously described in-liquid assay using *L. monocytogenes* as indicator. *L. lactis* 41FLL3 was grown in M17 broth at 30 and 37 °C and in the same medium fortified with 2% (*w/v*) fructose and 2% *(w/v*) glucose in order to improve Nisin Z production.

Sakacin P purification: The solution obtained from *L. sakei* I151 was loaded on a 5 mL HiTrap SP FF cation exchange column, previously equilibrated with 20 mM sodium acetate pH 4.2 (start buffer), using a flow rate of 5 mL/min. After sample loading, the column was washed with 25 mL of start buffer and the bacteriocin was eluted with 25 mL of elution buffer (start buffer fortified with NaCl 1M). Two mL fractions were collected and their antimicrobial activity was measured by the previously described in-liquid assay using *L. monocytogenes* as indicator. The fractions displaying antimicrobial activity were pooled and supplemented with 10% (*w/v*) ammonium sulphate as anti-chaotropic salt and immediately filtered to remove turbidity (0.45 μm filter). The obtained solution was applied to a 1 mL HiTrapOctyl FF hydrophobic interaction column, previously equilibrated with 20 mM sodium acetate pH 4.2 containing 10% (*w/v*) ammonium sulfate, using a flow rate of 1mL/min. The column was washed with the same buffer and the bacteriocin was then eluted with ethanol/start buffer 70:30. One mL fractions were collected and their anti-*Listeria monocytogenes* activity was evaluated by the in-liquid assay.

Purified nisin and sakacin were checked by Tricine-SDS-PAGE as described by Schägger (2006) [43] in Mini-PROTEAN Tetra Cell with Mini-Protean Tricine precast gels. Samples were quantified by the Bradford assay [41] and diluted in the sample buffer (12% SDS (*w/v*), 6% mercaptoethanol (*v/v*), 30% glycerol (*w/v*), 0.05% Coomassie Blue, 150 mM Tris-HCl pH 7) in order to load into the gels 5 µg of proteins. Molecular weight markers were from PageRuler Low range unstained protein ladder. The gels were stained with colloidal Coomassie Blue [44,45].

### 2.7. Evaluation of the Proteolytic Ability Towards Caseins

In order to identify the decrypted peptides from caseins, the strains were grown at 30 °C in a Chemical Defined Medium (CDM) as reported previously [46], with some differences; 100 time less valine, isoleucine, leucine amino acids and enriched with 0.5 g/L bovine caseins. Samples were collected in the late exponential phase by centrifugation at 4000× *g*, for 20 min, at 4 °C. The supernatants were filtrated using Vivacel 250 membrane filter (cut-off 5 kDa) and subsequently lyophilized. In order to screen the strain for their hydrolytic potential towards caseins, lyophilized samples were resuspended in water and peptide release was calculated by measuring the primary amines (–NH_2_) released, using a microplate analysis based on the reaction of ortho-phthaldialdehyde (OPA) and DTT, following the protocol reported by Deglaire et al. (2019) [47]. Only the strains with –NH_2_ released up to 300 mg/L were considered for the MS analysis.

### 2.8. Protein Identification of SDS-PAGE Bands and Analyses on Decrypted Peptides by MALDI-TOF/TOF

Protein digestion from SDS-PAGE bands was carried out as previously described by Nebbia et al., (2019) [48]. The resulting peptide mixtures were analyzed by a Ultraflex III MALDI-TOF/TOF instrument, as already described [49]. Manual/visual evaluation of the mass spectra was performed using Flex Analysis software. MASCOT software (www.matrixscience.co) version 2.4.0 was used for the protein identification against UniProtKB database, with the taxonomy restriction to Other Firmicutes. The MASCOT search parameters were: “trypsin” as enzyme, allowing up to 3 missed cleavages, carbamidomethyl on cysteine residues as fixed modification, oxidation of methionine as variable modifications. The peptide mass tolerance was 30 ppm.

For the analyses of the decrypted peptides from caseins, lyophilized samples containing peptide mixture were dissolved in 25 mM NH_4_HCO_3_, at the concentration of 10 mg/mL and analyzed by a Ultraflex III MALDI-TOF/TOF instrument as already mentioned. The spectra were searched with MS-non-specific software (http://prospector.ucsf.edu/prospector/mshome.htm) using an in-house bovine casein database (UniProt accession number: P02668, P02666, P02663 and P02662). The peptide mass tolerance was set at 30 ppm.

### 2.9. In Silico Analyses of the Identified Peptides

The bioactivity potential of the identified peptide was searched by the MBPDB online database (http://mbpdb.nws.oregonstate.edu/) [50]. The similarity threshold was set to 100% and the amino acid scoring matrix was set to identity.

### 2.10. Statistical Analyses

Statistical analyses were performed using R software version 4.0.2. Results were analysed using the one-way ANOVA. Normality of the residuals was assessed by means of Shapiro–Wilk’s test. Where significance was assessed, post hoc tests were conducted using Tukey’s multiple comparison post hoc test. Differences were considered significant at a minimum *p* value of 0.05.

## 3. Results and Discussion

### 3.1. Screening for Anti-Listeria Active LAB

Fourteen LAB strains isolated from both cheese and fermented sausages and one strain, *L. lactis MG1363,* belonging to the culture collection of LISBP (Toulouse, France) were grown in liquid media. Once reached the end-logarithmic phase, cultures were centrifuged and the supernatant tested against a culture of *L. monocytogenes* to detect a possible inhibitory action. As shown in Table 1, only four strains were active against the target bacterium, namely *L. sakei* I151, *L. lactis* SN12, *L. lactis* SN17, and *L. lactis* 41FLL. Further investigations have been performed on these 4 strains. Twenty-one LAB isolated from brewer’s grains, all belonging to the genera *Lactobacillus* and *Pediococcus* were able to inhibit *Listeria monocytogenes* growth in vitro [51]. In agreement with this finding, a recent report describes the efficacy of two LAB species, namely *Lactobacillus plantarum* and *Pediococcus pentosaceus* in reducing the amount of *Listeria monocytogenes* on cantaloupes whose consumption is cause of severe illnesses, hospitalization, and deaths [10]. A bacteriocinogenic strain of *Enterococcus* has been reported to be able to reduce *Listeria monocytogenes* contamination of meat especially at the presence of NaCl and ascorbic acid [52]. In addition, a review by [53] illustrates that control of listeriosis in meat can be obtained especially by means of LAB bacteriocins.

### 3.2. Screening of LAB for Bacteriocin Production and LDH Activity 

Based on the hypothesis that a specific inhibitory activity on *L. monocytogenes* can be achieved by means of antimicrobial peptides, namely bacteriocins, bacterial supernatants from the four interfering strains were treated with proteinase K and the interfering activity measured again. Actually, for two of the four strains inhibiting *L. monocytogenes* growth, namely, *L. sakei* I151 and *L. lactis* 41FLL, the interfering activity was lost after protease treatment, thus suggesting that growth inhibition can be ascribed to proteinaceous molecules (Table 1). Excluding any bacteriocin production for the strains retaining antimicrobial activity after proteinase treatment (*L. lactis* SN12 and *L. lactis* SN17), and assuming that acidification can prevent *L. monocytogenes* growth [54], we evaluated medium pH and lactate dehydrogenase (LDH) activity. As shown in Table 1, time-course acidification has been comparatively evaluated for the four interfering LAB and for 2 negative control strains (*L. lactis* MG1363 and *L. acidophilus* 41R). *L. lactis* SN12 and *L. lactis* SN17, showed an increased acidification after 24 h, reaching a pH of about 4. On the contrary, *L. sakei* I151 and *L. lactis* 41FLL displayed an acidification profile very similar to the control strains: maintaining a pH around 5.0 after 24 hrs. To ascertain if the pH lowering observed for *L. lactis* SN12 and *L. lactis* SN17 was due to increased lactic acid accumulation and hence to an enhanced catalysis, LDH activity was assessed. The data referred in Table 1 underline that the two strains responsible of significant pH lowering both possess a catalytically very efficient LDH as compared to the other tested strains. Furthermore, the two interfering strains (*L. sakei* I151 and *L. lactis* 41FLL) which induced a slight acidification, showed lower values of catalytic activity than *L. lactis* SN12 and *L. lactis* SN17, as expected. Therefore, the latter belong to the so-called high lactic acid producers that inhibit the growth of pathogenic bacteria by means of environment pH lowering described by Lado and Yousef [54].

Based on the assumption that the lost antibacterial activity detected for *L. sakei* I151 and *L. lactis* 41FLL following protease treatment is linked to specific antibacterial compounds, namely bacteriocins, further analyses aimed to partially characterize and purify the inhibiting molecules were performed on these two strains.

### 3.3. Searching for Specific Antimicrobial Molecules: Bacteriocin Investigation

**Figure 1:**
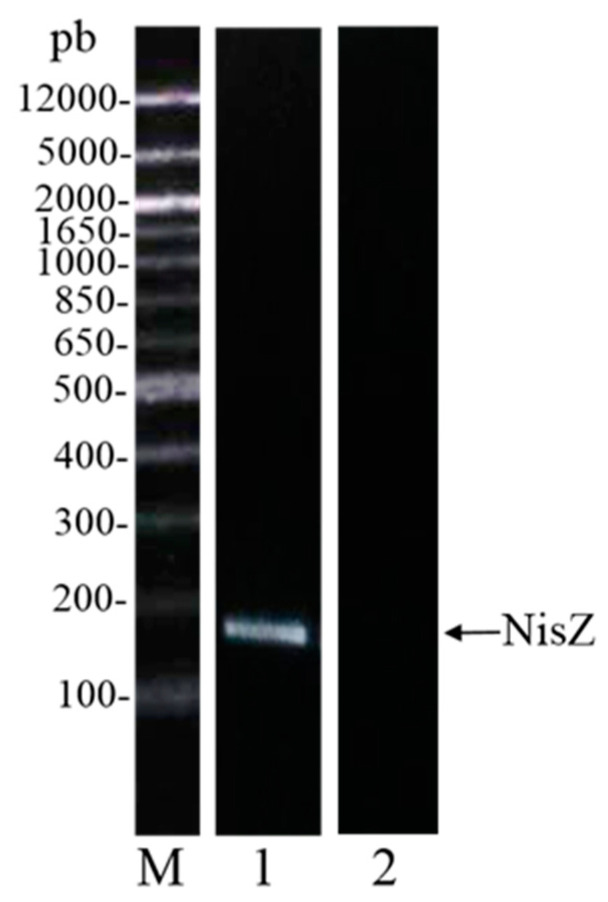
Identification of the NisZ gene at 174 bp, for the strain *L. lactis* 41FLL. M = molecular markers, Lane 1 = *L. lactis* 41FLL, Lane 2 = negative control.

First, we excluded the presence of folded proteins (bacteriolysins) by treating the supernatants of *L. lactis* 41FLL and *L. sakei* I151 at 90 °C. This procedure did not affect the inhibitory potential of cell-free supernatants (data not shown) thus supporting evidence for a peptide molecule. Considering that *L. lactis* is a good nisin producer, the genes for nisin were targeted after PCR in the strain *L. lactis* 41FLL. As shown in Figure 1, the NisZ band is clearly detectable in agarose gel. The most studied bacteriocin produced by LAB is Nisin A, which has been approved by the World Health Organization as food preservative. Nisin Z, a natural variant of Nisin A, was firstly isolated from *L. lactis* ssp. *lactis* NIZO 22186 by Mulders and co-workers (1991) [55]. Nisin Z differs from Nisin A for the presence of an asparagine instead of a histidine in position 27. This substitution has no effect on the antimicrobial activity, although the increased solubility of Nisin Z at pH 7.0 can offer greater potential applications at neutral pH [56].

Besides being employed directly as biocontrol agents, LAB can also be exploited as cell factories for the production of antimicrobial molecules. Hence, purification strategies for Nisin Z were set up. Supernatants from *L. lactis* 41FLL3 cultures were fractionated by a cation exchange chromatography. The fraction displaying the highest inhibitory attitude (55%) was quantified by the Bradford assay. The total produced nisin Z content was 2.32 mg/L (Table 2). Since it has been reported that environmental factors can account for better production of interfering molecules [57], in order to improve the bacteriocin yield, some culture parameters were changed. When the bacterial growth temperature was enhanced from 30 to 37 °C, the anti-*L. monocytogenes* activity of the purified Nisin Z increased from 55% up to 65%, although the purification yield was the same as the one measured at 37 °C (around 2 mg/L). Then, both glucose and fructose (in addition the M17 intrinsic lactose) were tested as sugar source for bacterial growth at 37 °C, because the importance of the sugar substrate in affecting bacteriocin production was previously described in the literature [58]. Both sugars were able to enhance the anti-*Listeria* activity of the 37 °C grown-cultures reaching around 95% of inhibition on *L. monocytogenes* growth. Furthermore, a remarkable enhancement of Nisin Z production was also assessed for both fructose- and glucose-fortified cultures, reaching 14.95 mg /L and of 10.55 mg/L of Nisin Z respectively.

**Figure 2:**
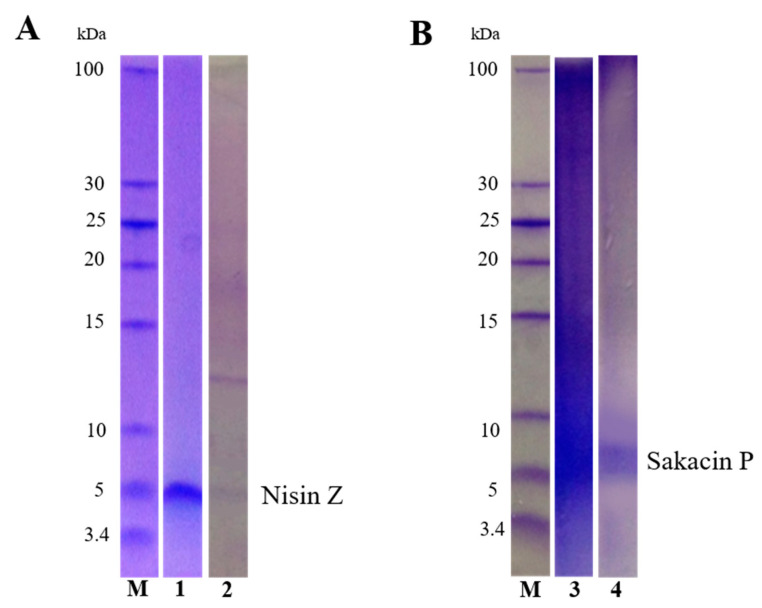
Tricine-SDS-PAGE Coomassie stained gel of purified Nisin Z and Sakacin P. Panel (**A**): antimicrobial fractions of *L. lactis* 41FLL3 grown in M17 enriched with fructose (2% *w/v*) (1) and with glucose (2% *w/v*) (2). Panel (**B**): antimicrobial fraction of *L. sakei* I151 after purification with cation exchange column (3) and hydrophobic interaction column (4). M: marker.

The effectiveness of Nisin Z purification was evaluated by Tricine SDS-PAGE (Figure 2, panel A). As shown in Figure 2 (Panel A-1) the fraction purified from the fructose-enriched cultures is visualized as a single band with the typical molecular weight of Nisin Z (4.5 kDa). The fractions purified from the glucose-enriched cultures (Figure 2, Panel A-2) showed an additional band at a molecular weight of about 13 kDa. To confirm the presence of Nisin Z in the lower MW band and to identify the protein contained in the second band, a MS analysis was performed. The band at lower molecular weight was identified as Nisin Z (UniProt entry P29559), the higher molecular weight band as the phosphocarrier protein HPr (UniProt entry P29559), a membrane transporter belonging to the PTS (phosphotransferase) system which is involved in sugar uptake and whose expression is generally induced by glucose [59].

The enhanced abundance of Nisin Z under specific growth conditions (37 °C, presence of glucose or fructose) is in agreement with data indicating that environmental factors can modulate the biosynthesis of this bacteriocin. Actually, nisin synthesis is regulated by a two-component regulatory system made up of the membrane-bound histidine kinase sensor protein NisK and the regulator NisR [60]. In particular glucose-inducing effect on bacteriocin production has been demonstrated in *Enterococcus* [61], and in *L. lactis* as well, whereas fructose-inducing effect was observed in *Pediococcus* [62].The high yield of nisin Z obtained in these modified growth conditions, together with the efficacy of a simple, cheap and quick one-step purification render this process potentially suitable for industrial applications. Actually, among antibacterial compounds, bacteriocins [34] are the most promising for industrial use. In fermented food, they can be produced in situ as a consequence of the bacterial metabolism. Alternatively, they can be added directly to food in a semi-purified form, however in this case their antimicrobial activity may be lost due to inactivation by food components, such as proteases and lipids [63]. More recently, the possibility to immobilize bacteriocins directly into the food packaging started to be explored since the polymer can protect bacteriocins from inactivation [63,64]. Active packaging was defined as “a type of packaging that changes the condition of the packaging to extend shelf-life or improve safety or sensory properties while maintaining the quality of the food” (European FAIR-project CT 98-4170) [65]. The concept of active packaging has been introduced as a response to the current consumer demands of healthy food (avoiding use of preservatives, high sugar, high salt) and in agreement with market trends aimed to counteract energy waste by the continuous cold-chain need [66]. Several studies verified the effectiveness of antimicrobial packaging material in inhibiting the development of a microbial strain inoculated into food [67,68]. Successful bacteriocin-functionalized packaging is still not widely available due to problems linked to the immobilization procedure that must ensure bacteriocin diffusion into polymers and gradual migration of the antimicrobial compound into food, without loss of activity [1] over time, during transport and storage of the end-products [69]. However, the true bottleneck is the difficulty to have high amounts of purified bacteriocins. Therefore, the highly active Nisin Z, directly purified from the culture medium, could be further immobilized into food packaging according to a new and challenging technology [70]. The use of antimicrobial films containing nisin Z could improve the quality, microbial safety and shelf-life of food products. In the literature, there are some reports dealing with the potential efficiency of nisin-based active packaging. A nisin-containing cellophane coating reduced viable counts of total aerobic bacteria in fresh veal meat stored at 8 °C [71], and an active packaging obtained from nisin-treated film inhibited *Micrococcus luteus* ATCC 10240 in broth as well as in raw milk and pasteurized milk during storage [72]. Finally, one of the advantages of bacterial-derived antimicrobial agents is their low induction of acquired resistance phenomena: nisin itself, is in use since 1953, and no (or very few) resistant strains have been described [30].

For *L. sakei* I151, sakacin was hypothesized as the most abundant and most frequently produced bacterocin. Conversely with what observed in *L. reuteri* that can produce different interfering molecules ranging from 100 to 2700 Da (see introduction) and in *L. lactis* where a large variety of bacteriocins occur such as lactococcins [73], lacticin [74] and nisin [60], *L. sakei* mainly produces sakacins. The first discovered was sakacin A [75] but later other very similar bacteriocins like sakacin B [76], sakacin K [77], sakacin G [78], the chromosomally-encoded sakacin T and sakacin X [79], sakacin P [80], sakacin LSJ618 [81] and sakacin C2 [82] have been described.

Sakacins are class IIa bacteriocins characterized by the amino acid sequence motif YGNGVXCXXXXCXV (in which X is any amino acid) in the hydrophilic, cationic N-terminal “pediocin box” [83]. Even in this case, the possibility to purify sakacin for further immobilization into the food packaging was explored [65]. Hence, sakacin was purified from *L. sakei* I151 supernatants by a two-step method, coupling cation exchange with hydrophobic interaction chromatography. After the first purification step, the two fractions displaying the highest anti-*L. monocytogenes* activity (85% and 75%, respectively) were pooled and separated by Tricine-SDS-PAGE. As shown in Figure 2 (Panel B-3), the pooled fractions were not properly purified, and then were further submitted to hydrophobic interaction chromatography. As shown in the Panel B-4, we obtained an improved purification. The purified bacteriocin was identified by MS analysis as sakacin P (UniProt entry P35618). However, due to the low purification yield (1.4 mg/L) and lower antimicrobial activity (65%) as compared to the original fractions, and to the need of cheap, fast and simple methods of purifications for industrial applications, further projects on sakacin were dismissed. On the other hand, successful bacteriocin-functionalized packaging is still not widely available due to the difficulty to have high amounts of purified bacteriocins without loss of activity over time, during transport and storage of the food product [70]. In case, also partially purified fractions of active bacteriocins can be used, provided that high antibacterial activity is present. Actually, some studies confirmed the effectiveness of semipurified bacteriocins immobilized into functionalized antimicrobial packaging in inhibiting the development of microbial strains inoculated into food [66,67].

### 3.4. Evaluation of the Ability to Decrypt Antimicrobial Peptides

A further aim of this work was to assess if some of the 14 strains were able to obtain an indirect antimicrobial effect by decrypting antibacterial peptides from casein, being most of them of dairy origin. The objectives were: (i) evaluating the proteolytic activity towards casein; (ii) if this occurred, analyzing the resulting peptides by MS and; (iii) searching for bioactive peptides screening *online* in MBPDB peptide database. LAB are known for having an efficient and complex proteolytic system evolved during their adaptation to the milk ecological niche (and parallel involution of their amino acid synthetic capability), and therefore well-fitting with casein [1].

**Figure 3:**
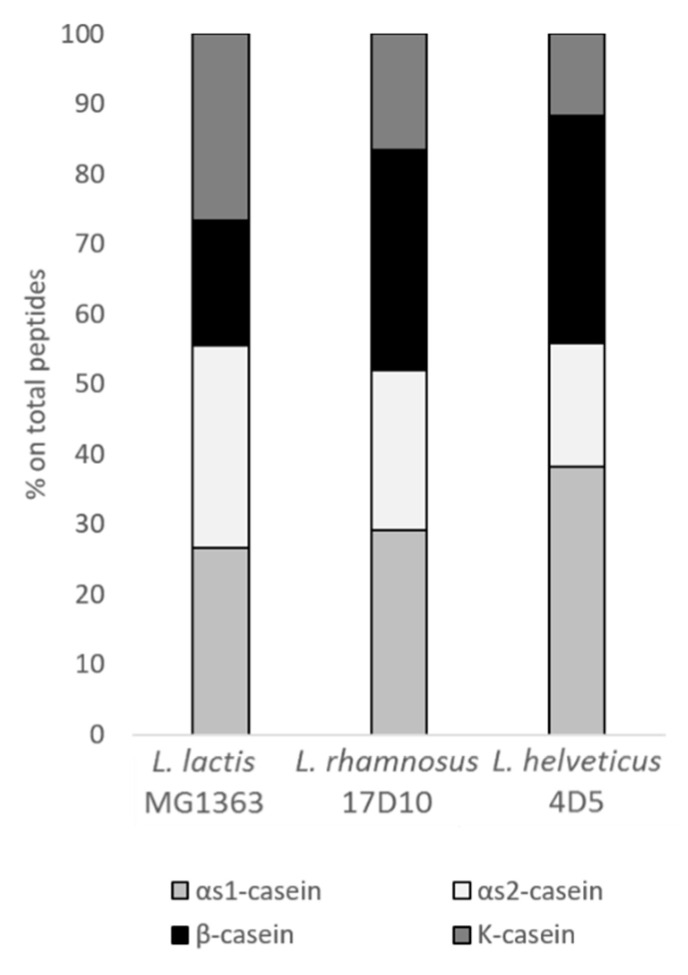
Percentage of casein-derived peptides harvested in the late exponential phase of *L. lactis* MG1363, *L. rhamnosus* 17D10 and *L. helveticus* 4D5 growth curves.

*L. lactis MG1363, L. rhamnosus 17D10* and *L. helveticus* 4D5 showed to be the highest peptide producers as determined by OPA quantification of the supernatants harvested in late exponential phase (data not shown). These three strains were able to hydrolyze all the casein added to the culture medium (Figure 3). In particular, the αs1- and αs2- caseins were the best targets for the three strains in study. For instance, for *L. helveticus* 4D5, 40% of the released peptides were originated from αs1-casein. As regards to the other two caseins, β-casein was mostly hydrolyzed by *L. rhamnosus 17D10* and *L. helveticus* 4D5, while k-casein and αs2-casein were mostly hydrolyzed by *L. lactis MG1363*. The list of the peptides identified by MS analysis is available as Table S1 in Supplementary Material.

**Figure 4:**
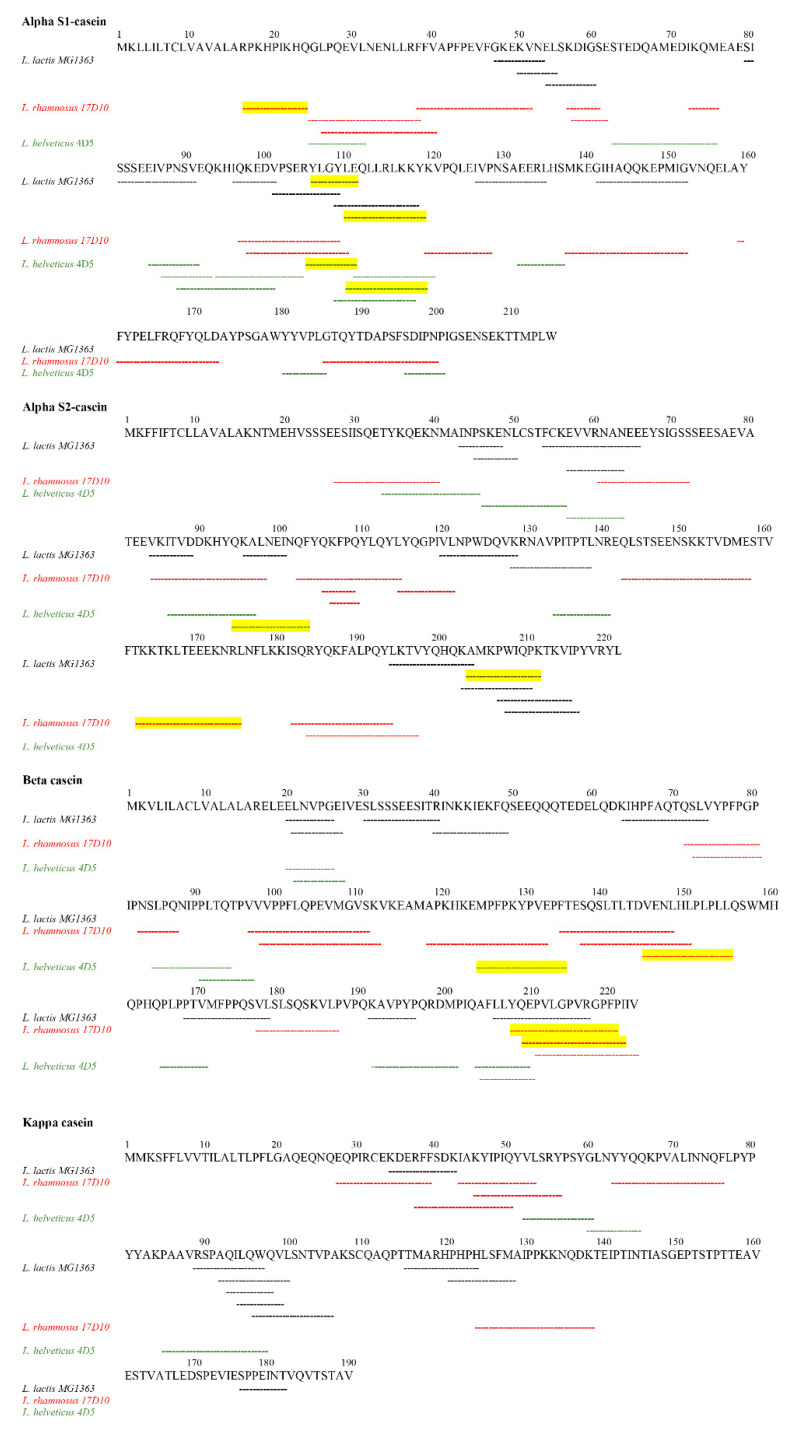
Peptide coverage of the four caseins digested by *L. lactis* MG1363 (black), *L. rhamnosus* 17D10 (red) and *L. helveticus* 4D5 (green). Peptides with established bioactivity are underlined in yellow.

As shown in Figure 4, all the three strains were able to hydrolyze even the central part of beta-casein reported to be the most resistant to proteolytic attack because of an alpha helix conformation [84]. Moreover, the same authors analyzed the proteolytic potential of some *L. helveticus* strains towards caseins and found that only certain domains, such as N-and C-terminus of αs1-casein that do not possess a true secondary structure, are suitable for hydrolysis. However, in the present investigation also the central part of the primary structure of αs1-casein was recognized by *L. helveticus* proteolytic system. Furthermore, the three strains displayed specificity of action since they released different peptides from αs1, αs2 and kappa caseins, suggesting that the target on the molecule was different. These results strongly indicate that the surface-bound proteases of these three strains are different in their mechanism of action. The selectivity of LAB proteolytic system towards specific amino acid sequences was previously described [85].

Peptides released by the strains in study were compared to known bioactive peptides reported in the data banks to find possible bioactivities. All the three strains were able to release bioactive peptides (Table 3). As far as antibacterial activity is concerned, four peptides showing 100% sequence identity to ascertained antimicrobial peptides were found, one of them being shared between *L. lactis* MG1363 and *L. helveticus* 4D5.

*L. lactis* MG1363 and *L. helveticus* 4D5 released the antimicrobial peptide LEQLLRLKKY from αs1-casein. This peptide was previously investigated by [87] that performed an in silico screening for the antibacterial activity of 248 peptides in bovine milk. LEQLLRLKKY was synthetized and tested in vitro against *E. coli* NEB 5, *B. subtilis* ATCC 6051 and *E. coli* ATCC 25922, showing inhibitory effect only towards the first two strains.

From the αs2-casein, *L. rhamnosus* 17D10 and *L. helveticus* 4D5 were able to decrypt other two antimicrobial peptides TKKTKLTEEEKNR and QKALNEINQF, respectively. Both peptides proved to have multifunctional biological properties, e.g., prolyl endopeptidase inhibition, ACE-inhibition, antioxidant and antimicrobial activity [90]. This behavior is due to overlapping regions called ‘‘strategic zones’’ able to exert the already mentioned biological activities. Moreover, a very flexible and dynamic structural conformation could further explain this multifunctional feature. As regards antimicrobial activity, peptide QKALNEINQF showed a higher effectiveness against all the tested strains (*Bacillus cereus*, *S. aureus*, *L. monocytogenes* and *Helicobacter pylori*) when compared to TKKTKLTEEEKNRL [90].

The last antimicrobial peptide (YQEPVLGPVRGPFPI) was decrypted by *L. rhamnosus* 17D10 from β-casein and was consistent with the well-known casecidin. It was isolated by Birkemo and co-workers [95] as naturally present peptide in bovine colostrum and its antimicrobial activity was tested against *E. coli* DH5α. This peptide was also obtained from caprine beta-casein after in vitro digestion with human gastrointestinal enzymes and its antibacterial activity was observed against *E. coli* K12 but not against *B. cereus* RT INF01 and *L. monocytogenes* [92].

In addition to peptides with antimicrobial effect, other informational peptides displaying different biological activities were found. This is of interest since some nutraceutical applications of bioactive peptides originating by microbial hydrolysis were described [35]. Five sequences showed a 100% of sequence identity to ACE-inhibitory peptides. Among them, three were released by *L. rhamnosus* 17D10 (RPKHPIKHQ, VENLHLPLPLL and QEPVLGPVRGPFPIIV), one by *L. lactis* MG1363 (AMKPWIQPK) and one by *L. helveticus* 4D5 (MPFPKYPVEP). ACE-inhibitors bioactive peptides from milk have gained attention in the formulation of new food products having antihypertensive properties and the strains able to produce ACE-inhibitor peptides are currently added to fermented dairy products [96]. The ACE-inhibitor peptide RPKHPIKHQ, released from αs1-casein by *L. rhamnosus* 17D10, was previously found by Saito et al. [89] in Gouda cheese and a significant hypotensive activity was demonstrated. The peptide VENLHLPLPLL, derived from β-casein hydrolysis by *L. rhamnosus* 17D10, was discovered by [91] after *L. helveticus* NCC2765 hydrolysis of skimmed milk and its resistance to pepsin and pancreatin digestion was demonstrated. Another peptide produced from β-casein by *L. rhamnosus* 17D10 was QEPVLGPVRGPFPIIV, already described as ACE-inhibitor peptide from Cheddar cheese by Lu et al. [93]. The AMKPWIQPK peptide, released from the αs2-casein by *L. lactis* MG1363 was previously found by Maeno et al. [88] after caseinate hydrolysis carried out by *L. helveticus* CP790. This peptide has the ability to induce a slightly decrease in the systolic blood pressure. *L. helveticus* 4D5 was able to decrypt the peptide MPFPKYPVEP from β-casein. As described by Hayes et al. [94], this peptide proved to have a strong ACE-inhibitory activity during in vitro tests and to be resistant to gastrointestinal digestive enzymes as well.

Finally, *L. lactis* MG1363 and *L. helveticus* 4D5 were also able to release the peptide YLGYLE from αs1-casein, which shows 100% sequence identity to an already known opioid peptide, previously identified from pepsin digestion of casein and named bovine α-casein exorphin [97]. This peptide is characterized by a negative charge conferring a weak affinity to μ- or δ-opioid receptors when compared to endogenous endorphins. More recently, Martínez-Maqueda and co-workers [86] suggested that its reduced opioid effect could be due to the hydrolytic activity of the intestinal peptidases that partially inactivate the peptide.

## 4. Conclusions

In the present study, besides the presence of naturally occurring high lactic acid producers, both bacteriocin and antimicrobial peptide (resulting from casein hydrolysis) producers have been detected from a pool of food-isolated LAB. Both these antibacterial compounds have gained great attention in recent years due to their low toxicity and large availability. From one side, they are appreciated in the food industry as natural preservatives, counteracting undesired contamination (resulting in food spoilage and shelf-life shortening) and controlling food-borne infections. This allows to reduce chemical preservatives and the amount of sugar and salt with excellent benefits for diabetics, obese and hypertensive subjects also supporting sustainable food storage with a lower need of the cold-chain. From the other side, their stability in blood and serum render them promising infection control agents and, after suitable modification or encapsulation or immobilization in polymeric matrices, they could represent a valuable strategy also for treating systemic infections. Furthermore, the present research has highlighted the potential of the proteolytic system of food-isolated lactic acid bacteria in decrypting also antihypertensive and opioid peptides useful as nutraceutical supplements to treat mild hypertension and children insomnia respectively. In the nutraceutical era, every effort directed to extend the number of natural compounds available is of interest. To reach these goals, the road is still long and winding and the present results, although circumscribed to *L. monocytogenes*, can add a further contribution in highlighting the richness of opportunities that LAB can offer, due to their plasticity, adaptability to changing conditions and because of their long history in food production.

## Figures and Tables

**Table 1:** Percentage of *L. monocytogenes* inhibition (pre- and post-proteinase K digestion), acidification profiles and LDH activity for the two control-strains not displaying antibacterial activity (*L. acidophilus* 41R and *L. lactis* MG1363) and for the four strains (*L. lactis* SN12, *L. lactis* SN17, *L. sakei* I151 and *L. lactis* 41FLL3) interfering with the growth of *L. monocytogenes*. Data represent means ± standard deviations (N = 3). Statistics: ANOVA *p* < 0.001 (***); *p* > 0.05 (ns). Tukey’s post hoc tests were performed and letters indicate significant differences between strains.

	*L. acidophilus* 41R	*L. lactis* MG1363	*L. lactis* SN12	*L. lactis* SN17	*L. sakei* I151	*L. lactis* 41FLL3	
***L. monocytogenes* inhibition** (%)	0.00 ± 0.11 ^a^	0.00 ± 0.13 ^a^	83.20 ± 3.02 ^b^	85.10 ± 2.30 ^b^	60.03 ± 1.21 ^c^	55.03 ± 2.02 ^c^	***
***L. monocytogenes* inhibition *post* proteinase K digestion** (%)	0.00 ± 0.10 ^a^	0.00 ± 0.11 ^a^	84.20 ± 2.51 ^b^	86.10 ± 1.30 ^b^	3.30 ± 0.81 ^c^	4.30 ± 1.19 ^c^	***
**Time** (h)	**pH**						
1	6.64 ± 0.05	6.64 ± 0.10	6.65 ± 0.10	6.71 ± 0.10	6.72 ± 0.10	6.56 ± 0.10	ns
2	6.61 ± 0.03 ^a^	6.55 ± 0.03 ^c^	6.63 ± 0.02 ^ad^	6.68 ± 0.01 ^bd^	6.69 ± 0.01 ^d^	6.57 ± 0.01 ^ac^	***
3	6.59 ± 0.03 ^a^	6.57 ± 0.01 ^a^	6.62 ± 0.03 ^abd^	6.66 ± 0.01 ^b^	6.65 ± 0.01 ^bd^	6.45 ± 0.02 ^c^	***
4	6.58 ± 0.02 ^a^	6.51 ± 0.02 ^d^	6.59 ± 0.02 ^a^	6.64 ± 0.02 ^b^	6.63 ± 0.02 ^ab^	6.35 ± 0.01 ^c^	***
5	6.55 ± 0.01 ^a^	6.37 ± 0.01 ^b^	5.93 ± 0.01 ^c^	6.61 ± 0.01 ^d^	6.60 ± 0.01 ^e^	6.24 ± 0.01 ^f^	***
6	6.52 ± 0.01 ^a^	6.24 ± 0.02 ^b^	4.95 ± 0.01 ^c^	5.02 ± 0.02 ^d^	6.59 ± 0.01 ^e^	6.09 ± 0.01 ^f^	***
24	5.29 ± 0.02 ^a^	5.00 ± 0.01 ^b^	4.17 ± 0.02 ^c^	4.21 ± 0.02 ^c^	4.96 ± 0.04 ^b^	4.98 ± 0.03 ^b^	***
**LDH activity** (U/mL of cell lysate/g protein)	10.02 ± 0.07 ^a^	9.98 ± 0.05 ^a^	32.02 ± 3.63 ^b^	29.07 ± 0.38 ^b^	15.26 ± 0.05 ^c^	14.33 ± 0.09 ^c^	***

**Table 2:** Percentage of L. monocytogenes inhibition by purified Nisin Z produced by *L. lactis* 41FLL3 grown at two different temperatures (30 and 37 °C) and with two different carbon sources (glucose and fructose) and Nisin Z purification yields (mg/L). Data represent means ± standard deviations (N = 3). Statistics: ANOVA *p* < 0.001 (***). Tukey’s post hoc tests were performed and letters indicate significant differences between strains.

	*L. lactis* 41FLL3 30 °C	*L. lactis* 41FLL3 37 °C	*L. lactis* 41FLL3 37 °C + Glucose (2% *w/v*)	*L. lactis* 41FLL3 37 °C + Fructose (2% *w/v*)	
***L. monocytogenes*** **inhibition (%)**	55.10 ± 1.19 ^a^	65.22 ± 1.09 ^b^	95.21 ± 1.89 ^c^	95.01 ± 1.60 ^c^	***
**Nisin Z purification yield** **(mg/L)**	2.32 ± 0.51 ^a^	1.90 ± 0.31 ^a^	10.55 ± 1.49 ^b^	14.95 ± 1.79 ^c^	***

**Table 3:** List of the identified bioactive peptides released by *L. lactis* MG1363, *L. rhamnosus* 17D10 and *L. helveticus* 4D5.

Sequence	Activity	Protein Precursor	Reference
***L. lactis* MG1363**			
YLGYLE	Opioid	αs1-casein (aa106-111)	[86]
LEQLLRLKKY	Antimicrobial	αs1-casein (aa110-119)	[87]
AMKPWIQPK	ACE-inhibitory	αs2-casein (aa204-2012)	[88]
***L. rhamnosus* 17D10**			
RPKHPIKHQ	ACE-inhibitory	αs1-casein (aa16-24)	[89]
TKKTKLTEEEKNRL	Antimicrobial	αs2-casein (aa163-176)	[90]
VENLHLPLPLL	ACE-inhibitory	β-casein (aa145-155)	[91]
YQEPVLGPVRGPFPI	Antimicrobial	β-casein (aa206-220)	[92]
QEPVLGPVRGPFPIIV	ACE-inhibitory	β-casein (aa209-224)	[93]
***L. helveticus* 4D5**			
YLGYLE	Opioid	αs1-casein (aa106-111)	[86]
LEQLLRLKKY	Antimicrobial	αs1-casein (aa110-119)	[87]
QKALNEINQF	Antimicrobial	αs2-casein (aa94-103)	[90]
MPFPKYPVEP	ACE-inhibitory	β-casein (aa124-133)	[94]

## Supplementary Materials

The following are available online at https://www.mdpi.com/2076-2607/9/1/65/s1, Table S1: List of peptides identified by MS analysis.
