# Supplementary material for: Antimicrobial Potential of Food Lactic Acid Bacteria: Bioactive Peptide Decrypting from Caseins and Bacteriocin Production
Source: Microorganisms. 2020 Dec 29;9(1):65. doi: 10.3390/microorganisms9010065 (PMC7824078; doi:10.3390/microorganisms9010065)
Supplement: Supplementary file 1 [file microorganisms-09-00065-s001.pdf]

Table S1. List of peptides identified by MS analysis.

Amino acid sequence	UniProt Entry	Starting position	Ending position	L. lactis MG1363	L. rhamnosus 17D10	L. helveticus 4D5
EKVN	P02662	48	53	X		
EKVNEL	P02662	50	55	X		
ELSKDIG	P02662	54	60	X		
SISSSEEIVPNSV	P02662	79	91	X		
VPSERYLGYV	P02662	101	109	X		
YLGYLE	P02662	106	111	X		X
YLEQLLRLKK	P02662	109	118	X		X
LEQLLRLKKY	P02662	110	119	X		X
IVPNSAEERL	P02662	126	135	X		
GIHAQQKEPMIG	P02662	141	152	X		
INPSKE	P02663	43	48	X		
PSKENL	P02663	45	50	X		
FCKEVVRNANE	P02663	54	64	X		X
EVVRNA	P02663	57	62	X		
VKITVD	P02663	84	89	X		
KALNEI	P02663	95	100	X		
VLNPWDQVK	P02663	120	128	X		
KRNAVPITPTL	P02663	128	138	X		
LKTVYQHQKA	P02663	195	204	X		
KAMKPWIQP	P02663	203	211	X		
AMKPWIQPK	P02663	204	212	X		
PWIQPKTKVI	P02663	207	216	X		
WIQPKTKVIP	P02663	208	217	X		
EELNVPG	P02666	19	25	X		X
ELNVPGE	P02666	20	26	X		X
SLSSSEESITRI	P02666	30	41	X		
RINKKIEKFQ	P02666	40	49	X		
HPFAQTQSLV	P02666	65	74	X		
PTVMFPPQSVL	P02666	168	178	X		
KAVPYP	P02666	191	196	X		
LLYQEPVLGPVR	P02666	206	217	X		
KDERFFSDK	P02668	34	42	X		
RSPAQILQW	P02668	89	97	X		
AQILQWQVL	P02668	92	100	X		
QILQW	P02668	93	97	X		
ILQWQ	P02668	94	98	X		
QWQVLSNTVP	P02668	96	105	X		
TMARHPHPH	P02668	115	123	X		
RHPHPHLSF	P02668	118	126	X		
SPPEINT	P02668	176	182	X		
RPKHPIKHQ	P02662	16	24		X	
GLPQEVLNENLLRF	P02662	25	38		X	
PQEVLNENLLRFFV	P02662	27	40		X	
FFVAPFPEVFGKEK	P02662	38	51			

SKDIG	P02662	56	60		X	
KDIGS	P02662	57	61		X	
IKQM	P02662	72	75		X	
IQKEDVPSERYLGY	P02662	96	109		X	
QKEDVPSERYLGYL	P02662	97	110		X	
KYKVPQLEIVP	P02662	118	128		X	
MKEGIHAQQKEPMIG	P02662	138	152		X	
FYPELFRQFYQLD	P02662	160	172		X	
TQYTDAPSFSDIPNPI	P02662	186	201		X	
ESIISQETYKQEKN	P02663	27	40		X	
NANEEEYSIG	P02663	61	70		X	
VKITVDDKHYQKAL	P02663	84	97		X	
QFYQKFPQYLQYL	P02663	102	114		X	
QKFP	P02663	105	108		X	
KFPQ	P02663	106	109		X	
LYQGPIVLNPWDQV	P02663	114	127		X	
QLSTSEENSKKTVDM	P02663	142	156		X	
TKKTKLTEEEKNRL	P02663	163	176		X	
ISQRYQKFALPQY	P02663	182	194		X	
SQRYQKFALPQYL	P02663	183	195		X	
QSLVYPFPGPIPNSLP	P02666	71	86		X	
SLVYPFPGPIPNSLPQ	P02666	72	87		X	
NSLPQNIPPLTQTPVV	P02666	83	98		X	
VVVPPFLQPEVMGVSK	P02666	97	112		X	
VVPPFLQPEVMGVSKV	P02666	98	113		X	
APKHKEMPFPKYPV	P02666	118	131		X	
FTESQSLTLTDVENL	P02666	134	148		X	
SQSLTLTDVENLHLP	P02666	137	151		X	
VENLHLPLPLL	P02666	145	155		X	
SVLSLSQSKVLP	P02666	176	187		X	
LYQEPVLGPVRGPFP	P02666	207	221		X	
YQEPVLGPVRGPFPI	P02666	208	222		X	
QEPVLGPVRGPFPIIV	P02666	209	224		X	
EQPIRCEKDERFF	P02668	27	39		X	
RFFSDKIAKYIPIQ	P02668	37	50		X	
IAKYIPIQYVL	P02668	43	53		X	
KYIPIQYVLSRY	P02668	45	56		X	
YYQQKPVALINNQF	P02668	63	76		X	
LSFMAIPPKKNQDKT	P02668	124	138		X	
GLPQEVL	P02662	25	31			X
STEDQAMEDIKQM	P02662	63	75			X
EIVPNSV	P02662	85	91			X
IVPNSVE	P02662	86	92			X
PNSVEQKHIQKE	P02662	88	99			X
QKHIQKEDVPS	P02662	93	103			X
AEERLH	P02662	131	136			X
YYVPLGTQYT	P02662	180	189			X

SDIPNPI	P02662	195	201			X
TYKQEKNMAINP	P02663	34	45			X
SKENLCSTFCK	P02663	46	56			X
TVDDKHYQKA	P02663	87	96			X
QKALNEINQF	P02663	94	103			X
PITPTLN	P02663	133	139			X
SLPQNIPPLTQT	P02666	84	95			X
PLTQTPV	P02666	91	97			X
MPFPKYPVEP	P02666	124	133			X
PLPPTVM	P02666	165	171			X
KAVPYPQRDM	P02666	191	200			X
QAFLLY	P02666	203	208			X
AFLLYQ	P02666	204	209			X
YVLSRYPSYG	P02668	51	60			X
GLNYYQ	P02668	60	65			X
PAAVRSPAQILQW	P02668	85	97			X
